# Spatio-Temporal Abnormal Behavior Prediction in Elderly Persons Using Deep Learning Models†

**DOI:** 10.3390/s20082359

**Published:** 2020-04-21

**Authors:** Meriem Zerkouk, Belkacem Chikhaoui

**Affiliations:** 1 Department of Computer Science, USTO-MB University, Oran 31000, Algeria; 2 LICEF Research Institute, Department of Science and Technology, TELUQ University, Montreal, QC 11290, Canada

**Keywords:** smart home, activity daily life (ADL), LSTM, CNN, autoencoder, abnormality detection

## Abstract

The ability to identify and accurately predict abnormal behavior is important for health monitoring systems in smart environments. Specifically, for elderly persons wishing to maintain their independence and comfort in their living spaces, abnormal behaviors observed during activities of daily living are a good indicator that the person is more likely to have health and behavioral problems that need intervention and assistance. In this paper, we investigate a variety of deep learning models such as Long Short Term Memory (LSTM), Convolutional Neural Network (CNN), CNN-LSTM and Autoencoder-CNN-LSTM for identifying and accurately predicting the abnormal behaviors of elderly people. The temporal information and spatial sequences collected over time are used to generate models, which can be fitted to the training data and the fitted model can be used to make a prediction. We present an experimental evaluation of these models performance in identifying and predicting elderly persons abnormal behaviors in smart homes, via extensive testing on two public data sets, taking into account different models architectures and tuning the hyperparameters for each model. The performance evaluation is focused on accuracy measure.

## 1. Introduction

The emerging Internet of Things (IoT) promises to create a world in which all the objects around us are connected to the Internet and communicate with each other with minimal human intervention. The crucial goal is to create a better world for human beings, in which the objects around us are context-aware, allowing them to respond to questions such as what we want, what we need and where we are. Smart homes are one of the main application domains of IoT, have received particular attention from researchers [1].

Smart homes provide a safe, secure environment for dependent people. They offer the ability (1) to track residents activities without interfering in their daily life; and (2) to track residents behaviors and monitor their health by using sensors embedded in their living spaces [2]. The data collected from smart homes needs to be deeply analyzed and investigated, in order to extract useful information about residents daily routines, in particular regarding specific activities of daily living. According to Reference [3], the training process can be distinguished into trained, training-free and trained-once. Therefore, this paper is interested in trained approach. 

Activity recognition [4], as a core feature of the smart home, consists of classifying data recorded by the different integrated environmental and/or wearable sensors into well-defined, known movements. However, dependent persons are often exposed to problems of various types that may cause them to perform activities of daily living incorrectly. Detecting abnormal behaviors is thus of great importance for dependent people, in order to ensure that activities are performed correctly without errors [5]. This will also ensure their safety and well-being. 

Detecting an anomaly in a person’s activities of daily living (ADL) is usually done by detecting nonconformities with their usual ADL patterns. Various authors have used classical machine learning algorithms to achieve this [6,7]. Tele-health care requires systems with high accuracy, low computational time and minimal user intervention because data are becoming larger and more complex [8]. Deep learning architectures provide a way to automatically extract useful and meaningful spatial and temporal features from raw data without the need for data labeling, which is time consuming, complex and error prone [9,10]. This makes deep learning models easily generalizable to different contexts. LSTM is a powerful deep learning model for sequence prediction and anomaly detection in sequential data [11]. The LSTM model is able to extract temporal features with long-term relationships. CNN has powerful spatial feature extraction capabilities and the ability to detect abnormality, which is considered as a classification problem. The advantage in the use of CNN with LSTM is to combine their capabilities in terms of spatial and temporal features extraction. Autoencoder learns a compressed input representation, which is recommended for the high dimensional data collected from smart home. Therefore, the motivation to select our deep learning models is due to their high performance accuracy in terms of abnormal behavior detection in different research areas [12,13].

This property is of great importance in smart homes in order to understand people’s behaviors, which change over time and particularly any deviations from normal execution of activities of daily living. 

In this paper, we investigate this variety of deep learning models such as LSTM, CNN, CNN-LSTM and Autoencoder-CNN-LSTM to identify and predict elderly people’s abnormal behaviors. The rationale of using deep learning models is fourfold: (1) the models are capable of handling multivariate sequential time-series data, (2) they can identify and accurately predict abnormal behavior in time-series data [14,15] and (3) they can automatically extract temporal and spatial features, from massive time-series data, making it easily generalizable to other types of data and (4) minimizing computation time. 

Therefore, the contributions of our paper can be summarized as follows:

1. Investigating a variety of deep learning models even models hybridization for automatic prediction of abnormal behaviors in smart homes.
2. Managing the problem of imbalanced data by oversampling minority classes for LSTM model in particular.
3. Conducting extensive experiments based on two public datasets to validate the proposed models.

The paper is organized as follows: Section 2 presents an overview of anomaly detection models and related work on machine learning algorithms. Section 3 presents materials and methods used to carry out our work. Section 4 shows the obtained results for each method/datasets. Finally, Section 5 discusses and highlights the obtained results.

## 2. Related Work

Tracking user behavior for abnormality detection has attracted considerable attention and is becoming a primary goal for some researchers [16]. Abnormal behavior detection approaches are based mainly on machine learning algorithms and specifically on supervised learning techniques [17]. Supervised classification techniques need labelled data points (samples) for the models to learn. This kind of classification entails training a classifier on the labelled data points and then evaluating the model on new data points. Thus, in the case of normal and abnormal classes, the model learns the characteristics of the data points and classifies them as normal or abnormal. Any data point that does not fit the normal class will be classified as an anomaly by the model. Various classification techniques have been applied for abnormal behavior detection. 

Pirzada et al. [18] explored the k-nearest neighbors algorithm (KNN), which works well to classify data into categories. Their method performs a binary classification in which activities are classified as good or bad, to distinguish anomalies in user behavior. The proposed KNN is applied to predict whether an activity belongs to the regular (good) or irregular (bad) class. Their method allows an unobtrusive use of sensors to monitor the health condition of an elderly person living alone.

Aran et al. [19] proposed an approach to automatically observe and model the daily behavior of the elderly and detect anomalies that could occur in the sensor data. In their proposed method, anomalies can be relied on to signal health-related problems. They therefore created a probabilistic spatio-temporal model to summarize daily behavior. Anomalies, defined as significant changes from the learned behavioral model, are detected and performance is evaluated using the cross-entropy measure. When an anomaly is detected, caregivers are informed accordingly.

Ordonez et al. [20] presented an anomaly detection method based on Bayesian statistics that identifies anomalous human behavioral patterns. Their proposed method automatically assists elderly persons with disabilities who live alone, by learning and predicting standard behaviors to improve the efficiency of their healthcare system. The Bayesian statistics are chosen to analyze the collected data and the estimation of the static behavior is based on the introduction of three probabilistic features: sensor activation likelihood, sensor sequence likelihood and sensor event duration likelihood. 

Yahaya et al. [21] proposed a novelty detection algorithm, known as the one-class Support Vector Machine (SVM), which they applied to the detection of anomalies in activities of daily living. Specifically, they studied an anomaly in sleeping patterns which could be a sign of mild cognitive impairment in older adults or other health-related issues. 

Palaniappan et al. [22] were interested in detecting abnormal activities of individuals by ruling out all possible normal activities. They define abnormal activities as randomly occurring, unexpected events. The multi-class SVM method is used as a classifier to identify the activities in the form of a state transition table. The transition table helps the classifier avoid states which are unreachable from the current state. 

Hung et al. [23] proposed a novel approach that combines SVM and Hidden Markov Model (HMM) in a homecare sensory system. Radio Frequency IDentification (RFID) sensor networks are used to collect the elder’s daily activities; an HMM is used to learn the data and SVMs are employed to estimate whether the elder’s behavior is abnormal or not. 

Bouchachia et al. [24] proposed a Recurrent Neural Network (RNN) model to address the problem of activity recognition and abnormal behavior detection for elderly people with dementia. Their proposed method suffered from the lack of data in the context of dementia. 

All of the aforementioned methods suffer from one or more of the following limitations:

1. The presented methods focus on spatial and temporal anomalies in user assistance. However, we note that abnormal behavior is not addressed in the smart home context;
2. These methods require feature engineering, which is difficult, particularly as data become larger;
3. The accuracy of abnormality identification and prediction is not sufficient; These points motivate us to propose methods, which seek to overcome these limitations and be useful for assistance in the smart home context.

## 3. Proposed Method

This section sets out the problem of abnormal behavior identification and prediction, describes different Neural Network (NN)-focused architectures and presents various hyperparameters for tuning the developed models.

### 3.1. Problem Description

Abnormality detection is an important task in health care monitoring, particularly for monitoring the elderly in smart homes. Abnormality detection consists of finding unexpected activities, variations in normal patterns of activities or patterns in data that do not conform to the expected behavior [25], because humans usually perform their ADLs in sequential manner. 

According to Zhu et al. [26], abnormalities can be classed as temporal, spatial or behavioral. Our work focuses on the behavioral class, because this kind of abnormality depends equally on time (when the activity is performed) and location (where the activity is performed). Each activity is defined by a sequence of sub-activities and if the person violates the expected sequence, that constitutes an abnormality.

### 3.2. Deep Learning for Abnormal Behavior Detection

Abnormal behavior detection is considered as a classification problem, in that the process entails using a time series as a model to predict future values based on previously observed values. It takes the order of the observations into account and uses models like Long Short-Term Memory (LSTM) recurrent neural networks, which have memory and can learn any temporal dependence between observations; the CNN model, which has a convolutional hidden layers that operate over a 1D sequence; and Autoencoder, which requires a dataset of sequences that are configured to read, encode, decode and recreate the input sequence. 

#### 3.2.1. LSTM

LSTM [27] is a recurrent neural network architecture whose principal characteristic is memory extension that can be seen as a gated cell, where gated means that the cell decides whether or not to store or delete information based on the importance it assigns to the information. Assignment of importance operates through weights, which are also learned by the algorithm. Simply put, this means that it learns over time which information is important. 

The LSTM architecture utilizes three types of layers: input, hidden and output. The hidden layers are fully connected to the input and output layers. A layer in LSTM is composed of blocks and each block has three gates: input, output and forget, which are all interconnected. These gates decide whether to let new input in (input gate), delete the information because it is not important (forget gate) or allow it to impact the output at the current time step (output gate).

As mentioned previously, our rationale for using LSTM is its ability to remember inputs over a long period, making it possible to remember data sequences. Abnormality detection aims to identify a small group of samples which deviate markedly noticeably from the existing data. That is why we have chosen LSTM to identify and accurately predict abnormal behavior from what is likely to be a long series of sequential data, given that people perform their ADL in a sequential manner. Less human intervention is thus required in the identification and prediction process.

The data must be reshaped to develop the LSTM input layer, which needs the input data to be 3-dimensional: that is, training sample, time step and features. For this layer, we added an activation function (ReLu). The dropout method [28] was used to avoid the overfitting problem in LSTM architectures and improve model performance. In our proposed model, the dropout is applied between the two hidden layers and between the last hidden layer and the output layer. We set the dropout at 20%, as recommended in the literature [29]. 

**Figure 1:**
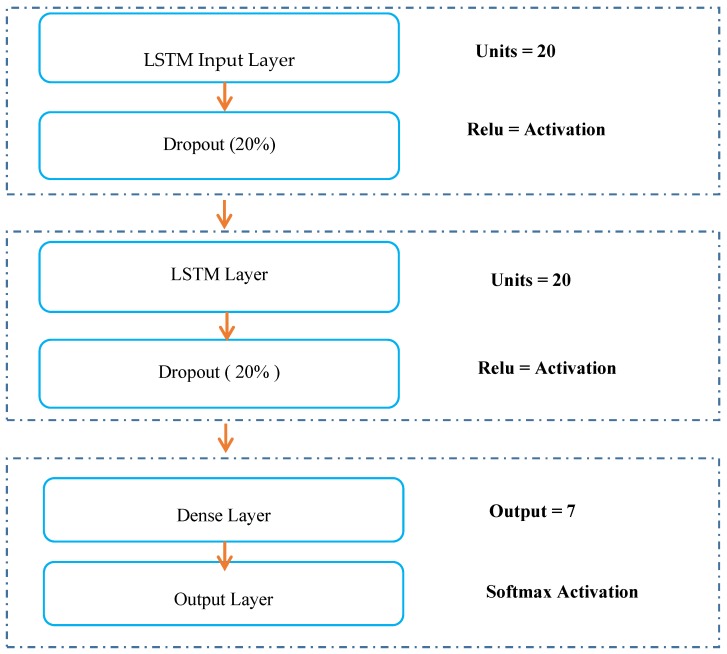
Long short term memory (LSTM) architecture development.

The last layer (dense layer) defines the number of outputs which represent the different activities and anomalies (classes). The output is considered as a vector of integers, which is converted into a binary matrix. The anomaly prediction is formulated as a multi-classification problem which requires the creation of (number of classes) output values, one for each class. Softmax is used as the activation function and categorical cross-entropy as the loss function. Figure 1 depicts the development of the LSTM architecture. 

#### 3.2.2. CNN

**Figure 2:**
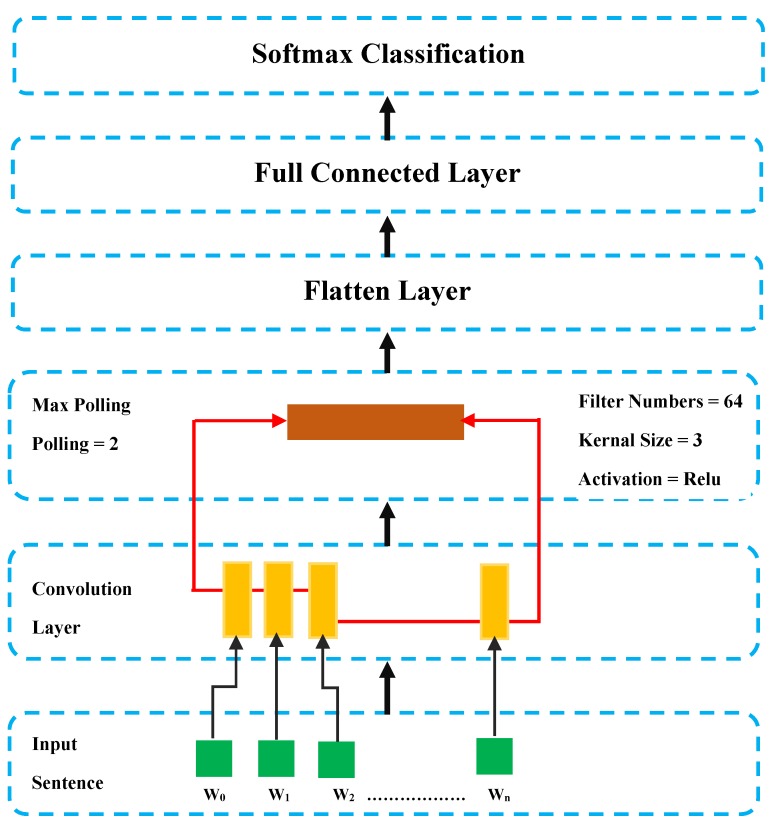
Convolutional neural network (CNN) architecture development.

CNNs are a class of neural networks generally used for image recognition and object classification. Our aim is to use CNN to identify abnormalities in time series, an area which is attracting attention, as they can learn directly from the raw time series data, extract features from sequences of observations, without domain expertise and manually engineer input features [30]. CNN development entails adapting the time series (temporal multidimensional 1D readings) by forming a virtual image, in a two-stage process. The first stage is a feature extractor, which learns features from raw data automatically. The second is a trainable fully-connected, which performs classification based on the features learned in the previous stage. We develop our CNN architecture based on a feature extractor which comprises a convolution layer, an activation layer, a pooling layer and a fully connected layer, each of which requires a feature map as input and as output [31], as described in Figure 2.

Our Convolution layer is a process that creates a feature map to predict the class probabilities for each feature by applying a filter (64) that scans the whole image, few pixels at a time. The shape of input to the convolution layer is (number of samples, number of timesteps, number of features per timestep). We add an activation function (Relu) that introduces non-linearity into the neural network and allows it to learn a more complex model. We use two convolutional hidden layer followed by a max pooling layer where max pooling (2) is a process that enables the CNN to detect an image when presented with modification. The convolution and pooling which can be repeated to have Conv3 or Conv4. The advantage of this approach is that we treat the 1D sensor reading as a 1D image, which is simple and easy to implement. After that, the Fully connected “flattens” the outputs generated by previous layers to turn them into a single vector that can be used as an input for the next layer, applies weights over the input generated by the feature analysis to predict an accurate label and generates the final probabilities to determine a class for the sequence 1D array. The output of these networks is often one or more fully connected layers that interpret what has been read and map this internal representation to a class value. Once the model is defined, it can be fitted in the training data and the fitted model can be used to make a prediction.

#### 3.2.3. Autoencoder-CNN-LSTM

An autoencoder [32] is a multi-layer neural network in which the desired output is the input itself. The aim of autoencoder is to learn more advanced feature representation in compressed representation to catch the most significant features of the training data [33].

The architecture is constructed on three layers: an input layer, a hidden layer and an output layer. To attain valuable features from the Autoencoder, the hidden units dimension is regularized to be smaller than the dimension of the input units. The framework usually includes the encoding and decoding processes. Given an input x, Autoencoder first encodes it to one or more hidden layers through several encoding processes, then decodes the hidden layers to obtain an output x.

**Figure 3:**
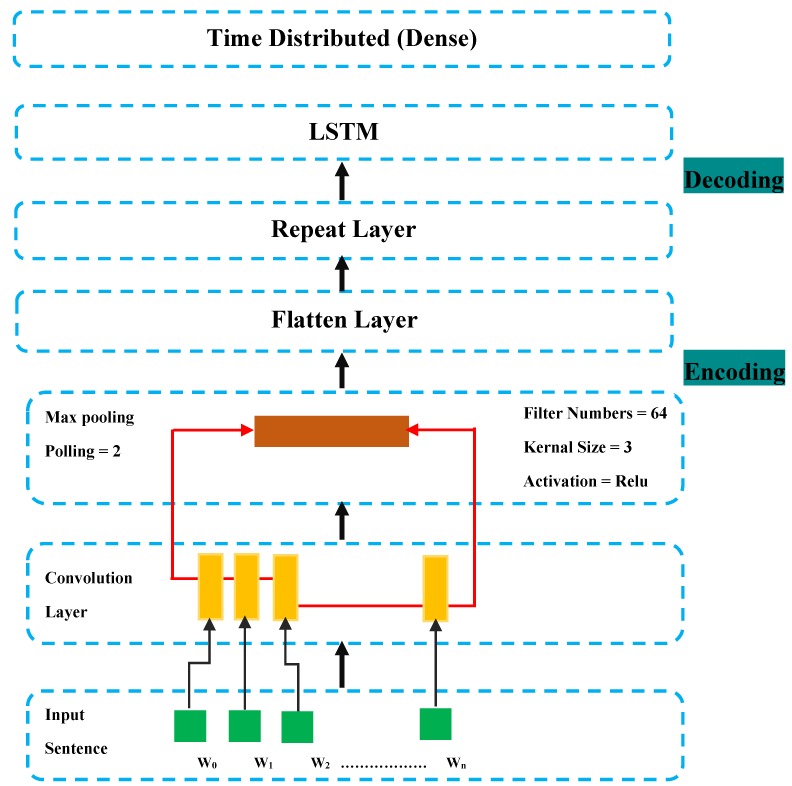
Autoencoder-CNN-LSTM Architecture development.

In this work, CNN and LSTM are integrated to Autoencoder to be considered as a classifier, the proposed framework is described in the Figure 3.

The developed architecture described in Figure 3 has CNN as encoder, RepeatVector (is used as the first layer of the decoder), LSTM layers as decoder and is dense with a TimeDistributed (Dense) layer.

The encoding developed by CNN, requires as input a 1D vector followed by pooling and flatten layer. The output of the layer is an encoded feature vector of the input data, which can be used as compressed data. The encoding is followed by a ReapeatVector, where its role is to replicate the feature vector and LSTM layer with a number of nodes and a TimeDistributed (Dense) layer.

## 4. Experiments 

For our experimental study to test our method’s ability to identify abnormalities, we selected two public datasets involving different types of abnormality. Because these datasets generally exhibit a problem of imbalanced classes, a Synthetic Minority Over-Sampling TEchnique (SMOTE) method was used to oversample our data. We then evaluated the classification method using hyperparameter tuning.

### 4.1. Datasets

#### 4.1.1. SIMADL Dataset

This research uses the SImulated Activities of Daily Living (SIMADL) [34] dataset generated by OpenSHS [35], an open-source simulation tool that offered the flexibility needed to generate residents’ data for classification of ADLs. OpenSHS was used to generate several synthetic datasets that include 29 columns of binary data representing the sensor values, where each binary sensor has two states, on (1) and off (0). The sensors can be divided into two groups: passive and active. The passive sensors react without the participant’s interacting explicitly with them. Instead, they react to the participant’s movements and positions. The sampling was done every second. Seven participants were asked to perform their simulations using OpenSHS. Each participant generated six datasets resulting in forty-two datasets in total. The participants self-labelled their activities during the simulation. The labels used by the participants were—Personal, Sleep, Eat, Leisure, Work, Other and Anomaly. The simulated anomalies are behavioral and are described in Table 1. Note that each user has his/her own behavioral abnormality to simulate where the abnormality kind is as a forget as shown in Table 1.

#### 4.1.2. MobiAct Dataset 

MobiAct is a public dataset version 2 [36], a smartphone placed in the pocket is used to collect the data. The participants are asked to perform different types of activities (such as walking, sitting, standing, ascending and descending stairs, jumping, jogging and biking). The Table 2 shows the different asked abnormality and the different kind of falls.

### 4.2. Imbalanced Data 

**Figure 4:**
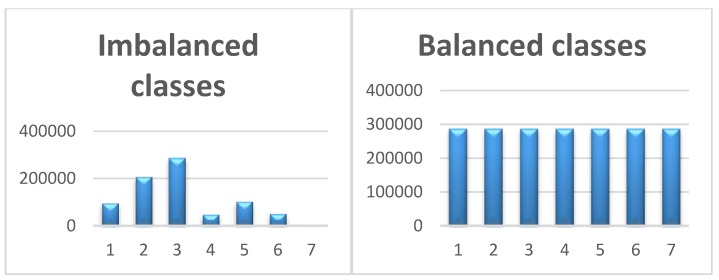
Imbalanced Classes Vs Balanced Classes.

The distribution of the classes representing the different ADL is not uniform, leading to imbalanced classes. This situation arises because of the rarity of abnormal behavior. This can be clearly seen in Figure 4, where the class “anomaly” constitutes a minority. We decided to tackle this problem in order to improve our classification performance. Dealing with imbalanced datasets requires strategies such as the use of oversampling techniques before providing the data as input to the LSTM model. The oversampling strategy involves augmenting the minority class samples to reach a balanced level with the majority class.

#### Oversampling

We deal with the abnormality (anomaly) detection problem as a supervised learning that involves correctly classifying rare class samples as compared to majority samples.

Anomalies constitute a minority in the whole set of behavior, which creates an imbalanced data problem. Therefore, we have to oversample our data, after which, we can classify correctly. 

To this end, a subset of data is taken from the minority samples as an example and new, synthetic, similar data points are created. These synthetic data points are then added to the original dataset and the resulting new dataset is used to train the classification models. The main approach to balancing classes is either to increase the samples of the minority class or decrease the samples of the majority class. In oversampling, we increase the minority class samples. This is done in order to obtain approximately the same number of instances for both classes, as demonstrated in Figure 4. Our rationale in using this strategy is to avoid overfitting. We used the SMOTE statistical method [37] to oversample our classes, as illustrated in Figure 4. We note that the x-axes indicate the number of classes and y-axes indicate the number of input data points.

### 4.3. Network Architectures and Hyper-Parameters Tuning Models with Different Datasets

The experiments were implemented in Python language using Keras library [37] with Tensorflow [38] to create the different LSTM, CNN and Autoencoders model architectures. Deep learning models are full of hyper-parameters and finding the best configuration for these parameters in such a high dimensional space is not a trivial challenge but there are some parameters, which are fixed for all architectures as shown in the Table 3.

Many experiments were run by varying LSTM networks architecture according to the hyperparameters as shown in Table 4 to find the suitable hyperparameters. To improve the LSTM performance, it is important to vary nodes, layers and epochs.

To compile and fit the model, we experimentally used the hyperparameters indicated in the Table 3.

Note that the datasets are sensible for imbalanced classes as described in sections above. The convenient architecture for the two dataset is 20 nodes, 4 layers and 10 epochs.

According to the CNN architecture described in the Section 3.2.2, we have experimented the framework with two datasets and the tuning of the CNN model requires varying the number of filters, size of kernel, pooling, number of layers and number of epochs as indicated in Table 5. 

All these variations according to the Table 5 is a clear improvement in the CNN to have the appropriate architecture. The appropriate architecture is attained with 64 filters, 5 kernels size, pooling 5, layers (2,3 and 4) and 10 epochs.

The CNN-LSTM model is an hybridization of LSTM model and CNN model seen in the sections above, is used to identify and extract significant temporal and spatial features from multivariate time series, taking advantage of the strength of CNN on feature extraction ability from raw data and the excellent time series processing ability of LSTM. In order to find the best configuration of CNN-LSTM, a hyperparameters tuning process is required but in such a high dimensional space, it is not trivial. Table 6 cites the different hyperparameters. 

It was very challenging to find the suitable architecture where the aim is to find the temporal and the spatial features. Finally, we reached the suitable architecture by these hyperparameters: 64 filters, kernel size (5), pooling (5), layers CNN (3), nodes (20), layers LSTM (1) and 10 epochs. Tuning the Autoencoder-CNN-LSTM model is same to the CNN-LSTM model by following the developed architecture seen in Figure 3.

## 5. Performance Metrics Analysis

As stated in the introduction section, the experimental study was carried out in order to identify and predict the abnormal behavior. To highlight the performance of the proposed methods, we consider the accuracy, the precision and the recall as performance measures for the different LSTM, CNN, LSTM_CNN and Autoencoder_LSTM_CNN models. The results of each method/datasets are summarized in Table 7; the presented results are interesting in several ways. In this section, we analyze our objective in term of abnormality detection where the captured abnormality is different from model to another. LSTM aims to capture temporal abnormal behavior sequences by incorporating memory cell to store temporal dependency and information. As stated in the Introduction section, the most important characteristic of deep learning is that it does not need any manually features extraction to learn and can easily learn a hierarchical feature representation from the raw data directly. 

The LSTM model clearly has an advantage over temporal information identification and prediction. The metrics shown in Table 7 indicate that LSTM model adequately captures the important features to boost detection abnormality accuracy. LSTM performs well in each dataset with an accuracy of 94% and 93% respectively. We also reported precision and recall measures which are shown in the Table 7. A comparison of LSTM with classic machine learning models was done in Reference [39]. 

CNN aims to capture spatial abnormal behavior sequences of time series based on an automatic features extraction. We reported in Table 7 the obtained results for the SIMADL and MobiAct datasets where CNN is experimented by testing an increasing number of layers (3-CNN and 4-CNN). The model performs well even by increasing the number of layers. The accuracy obtained by the CNN models in both datasets was 93% and 91% respectively. As shown in Table 7, the hybridization of CNN with LSTM is interesting, it has strong ability in terms of the extraction of temporal and spatial features automatically at the same time. According to the obtained accuracy, precision and recall, it was decided that the model gives the best performance in terms of abnormality detection. 

The hybridization of CNN with LSTM achieves an accuracy of 98% in the SIMADL dataset and 93% in the MobiAct dataset. This could be explained by the fact that temporal and spatial features are two important types of features in detecting abnormal behaviors. 

Autoencoder-CNN-LSTM provides additional support and a clear improvement for our problem in the compressed manner and it can be seen from Table 7 that it gives the best accuracy, precision and recall for only MobiAct dataset. In contrast, testing the model on SIMADL, accuracy of 84% was obtained. Therefore, we cannot generalize its usefulness. 

**Figure 5:**
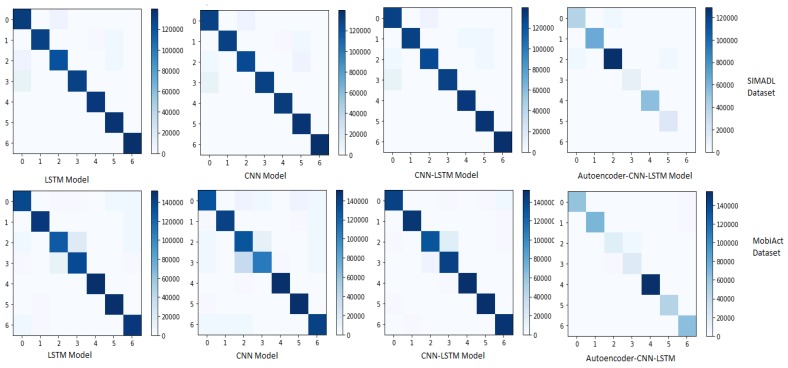
Confusion matrix (model/dataset).

In order to check if the obtained accuracy and precision were not misleading, we use a confusion matrix for each model and both datasets. LSTM, CNN and CNN-LSTM perform well when the datasets are oversampled by using SMOTE method as shown in Figure 5. 

## 6. Conclusions

This paper presents a comparative study of abnormal behavior prediction methods (LSTM, CNN, CNN-LSTM, Autoencoder-CNN-LSTM) based on neural networks. The presented methods identify and predict abnormal behaviors with a high degree of accuracy and require less user intervention in order to automate the identification and prediction process. Two public datasets are chosen to validate the methods performance.

The non-uniform distribution of classes generates an imbalanced classes problem which is addressed by applying the SMOTE method for oversampling the classes. LSTM, CNN and CNN-LSTM models are affected by this problem in particular. LSTM gives a good result for temporal extraction, CNN proves its ability to extract the spatial information in time series, the hybridization of CNN and LSTM is validated in the purpose to extract the temporal and spatial features. Another interesting manner to extract the features by compressing them in the unsupervised manner with Autoencoder-CNN-LSTM model, we have to improve it in order to generalize it. 

In future work, an analysis of users’ outdoor behavior could provide a fuller understanding of elderly people’s health and thereby improve their well-being.

## Figures and Tables

**Table 1:** Description of abnormality.

Participants	Description
Participant 1	Leaving fridge door open.
Participant 2	Leaving oven on for a long time.
Participant 3	Leaving main door open.
Participant 4	Leaving fridge door open.
Participant 5	Leaving bathroom light on.
Participant 6	Leaving tv on.
Participant 7	Leaving bedroom light on and wardrobe open.

**Table 2:** Description of fall abnormality.

Code	Activity	Description
FOL	Forward Laying	Fall Forward from standing using of hands.
FKL	Front-Knees-	Fall forward from standing, first impact on knees
SDL	Sideward-lying	Fall sideward standing
BSC	Back-sitting-chair	Fall backward while trying to sit on a chair.

**Table 3:** Fixed hyperparameters.

Parameters	Values
Activation	32, 64, 128
Optimizer [38]	2,3,5
Batch size	2,3,5

**Table 4:** LSTM Hyperparameters.

Parameters	Values
Nodes	20, 64, 100
LSTM layers	1, 2, 3, 4
Epochs	10, 50, 100

**Table 5:** CNN hyperparameters.

Parameters	Values
Filters	32, 64, 128
Kernel size	2,3,5
Pooling	2,3,5
CNN layers	1,2,3,4

**Table 6:** CNN-LSTM hyperparameters.

Parameters	Values
Filters	32, 64, 128
Kernel size	2,3,5
Pooling	2,3,5
CNN layers	1,2,3,4
Nodes	20, 32, 64,100
LSTM layers	1, 2, 3, 4
Epochs	10, 50, 100

**Table 7:** Accuracy by methods/datasets.

Datasets\Models	Metrics	LSTM	CNN	CNN-LSTM	Autoencoder-CNN-LSTM
SIMADL	Accuracy	94%	93%	98%	84%
	Precision	93%	90%	94%	80%
	Recall	93%	90%	94%	80%
MobiAct	Accuracy	93%	91%	93%	98%
	Precision	91%	90%	92%	93%
	Recall	91%	90%	92%	93%
